# FABP4 levels in hypothyroidism and its relationship with subclinical atherosclerosis

**DOI:** 10.3906/sag-1904-41

**Published:** 2019-10-24

**Authors:** Mürşide TAN, Hakan KORKMAZ, Hüseyin AYDIN, Duygu KUMBUL DOĞUÇ

**Affiliations:** 1 Department of Internal Medicine, Faculty of Medicine, Süleyman Demirel University, Isparta Turkey; 2 Division of Endocrinology, Department of Internal Medicine, Faculty of Medicine, Süleyman Demirel University, Isparta Turkey; 3 Department of Radiology, Faculty of Medicine, Süleyman Demirel University, Isparta Turkey; 4 Department of Biochemistry, Faculty of Medicine, Süleyman Demirel University, Isparta Turkey

**Keywords:** Hypothyroidism, carotid artery intima media thickness, fatty acid bindingprotein 4, atherosclerosis

## Abstract

**Background/aim:**

The aim of this study is to evaluate the relationship between serum fatty acid binding protein 4 (FABP4) levels and carotid intima media thickness (CIMT) in patients with hypothyroidism.

**Materials and methods:**

Forty subclinical hypothyroidism patients, 40 overt hypothyroidism patients, and 40 healthy controls were enrolled in the study. Blood pressure, body mass index, CIMT, fasting blood sugar, creatine, alanine aminotransferase, lipid parameters, insulin, free thyroxine, triiodothyronine, thyroid-stimulating hormone (TSH), thyroid peroxidase antibody (anti-TPO), thyroglobulin antibody (anti-TG), high-sensitivity C-reactive protein (Hs-CRP), and FABP4 levels of all participants were measured.

**Results:**

Serum FABP4 levels were significantly higher in patients with subclinical and overt hypothyroidism than healthy controls (HCs) (P = 0.044 and P *= *0.014, respectively). There was no significant difference in terms of FABP4 levels between patients with subclinical and overt hypothyroidism (P* = *0.641). Serum TSH levels and serum FABP4 levels were positively correlated (r = 0.201, P = 0.039). CIMT was found to be higher in patients with subclinical and overt hypothyroidism than in HCs (P = 0.042 and P < 0.001, respectively). No correlation was found between CIMT and FABP4 levels (r = 0.038, P = 0.702). There was a positive correlation between CIMT and TSH, anti-TPO, anti-TG, triglycerides (TG), and total cholesterol levels. It was found that high TG levels were an independent factor that increased CIMT (r = 0.382, r2 = 0.146).

**Conclusion:**

In patients with subclinical and overt hypothyroidism, the level of FABP4 increases and this increase is correlated with the increase in TSH level. It is thought that FABP4 does not play a role in atherosclerosis development in patients with hypothyroidism without metabolic disorder.

## 1. Introduction

The risk of developing cardiovascular disease (CVD) increases in patients with hypothyroidism [1]. One of the factors responsible for CVD in hypothyroidism is the development of endothelial dysfunction and reduced response to nitric oxide (NO) [2,3]. Although it is not clearly known which mechanisms lead to this condition, symptomatic improvement after thyroid hormone replacement has been observed [4,5]. In addition, hypothyroidism also leads to the development of dyslipidemia, hypercholesterolemia, weight gain, insulin resistance, coagulation disorders, inflammation, and diastolic hypertension (HT). The development of these disorders can also contribute to the incidence of CVD in hypothyroidism [6–12].

Fatty acid binding protein 4 (FABP4) is a newly described adipocytokine that is released from adipocytes and macrophages [13] and is associated with the development of metabolic syndrome (MetS), insulin resistance, and increased inflammation. The level of FABP4 has been suggested to be a novel marker that can strongly predict the risk of MetS development and CVD [14]. Regression in atherosclerotic lesions has been reported after treatment with a FABP4 inhibitor [15]. FABP4 expression is strongly induced during the differentiation of adipocytes as well as the differentiation of macrophages from monocytes. The induction of these cells is regulated by proinflammatory stimuli [14], which in turn are known to be regulated by triiodothyronine (T3) [16].

Although both thyroid hormones and FABP4 levels are associated with CVD development, the relationship between the two has not been evaluated to date. The identification of such a relationship will provide a better understanding of the mechanism by which hypothyroidism increases the risk of CVD. Clarifying the etiopathogenesis of this will guide future studies on targeted molecular therapy. In the current study, we aimed to evaluate the effect of FABP4 on the development of CVD in patients with hypothyroidism.

## 2. Materials and methods

This study was approved by the local ethics committee. All patients to be included in the study were informed verbally and in writing about the research and informed consent forms were obtained from those who agreed to participate in the study.

### 2.1. Selection of the subjects to be included in the study

The study group consisted of 40 patients between the ages of 18 and 65 years (3 males, 37 females) who were admitted to our clinic with subclinical hypothyroidism (SH), 40 patients (9 males, 31 females) between the ages of 18 and 65 years who were admitted to our clinic with overt hypothyroidism (OH), and 40 people (3 males, 37 females) with no systemic disease who formed the healthy control (HC) group. The patients with SH had thyroid-stimulating hormone (TSH) levels greater than 4.94 µIU/L with normal free thyroxine (fT4) levels. Patients with OH had TSH of >4.94 and low fT4 levels.

Patients with inflammatory diseases (acute infectious diseases, malignancies, inflammatory rheumatic diseases); pregnant patients; those with chronic kidney disease, diabetes mellitus, MetS, HT, and acute or chronic liver disease; and patients using glucocorticoids and nonsteroidal antiinflammatory drugs were excluded from the study.

### 2.2. Anthropometric and blood pressure measurements

Blood pressure measurements were carried out for both brachial arteries after the patient had rested for at least 5 min in sitting position prior to the measurements. Body weight and height measurements of all participants were recorded and body mass index (BMI) was calculated by dividing the body weight by the square of the height (kg/m2).

### 2.3. Biochemical analysis

Fasting blood glucose (FBG), creatine, alanine aminotransferase (ALT), high-density lipoprotein-cholesterol (HDL-C), triglycerides (TG), low-density lipoprotein-cholesterol (LDL-C), total cholesterol (TC), free triiodothyronine (fT3), free thyroxin (fT4), thyroid-stimulating hormone (TSH), and insulin levels were measured by using the electrochemiluminescence method with an Abbot Aeroset chemical commercial kit (Abbot Park, IL, USA). Insulin resistance was calculated by using the homeostasis model assessment (HOMA = (FBG × insulin)/405)) [17]. Antithyroid peroxidase (anti-TPO) and antithyroglobulin (anti-TG) levels of the patients with SH and OH were also measured. FABP4 and high-sensitivity C-reactive protein (Hs-CRP) levels were measured by Biosensitive DPC (USA) brand Immulatory 2000 device using compatible commercial kits.

### 2.4. Carotid intima media thickness measurement

The carotid intima media thickness (CIMT) of each subject was measured by using a 7.5–13.5 MHz multifrequency linear array probe with B-mode ultrasonography (USG) and duplex Doppler examination (Shimadzu, Japan). All USG examinations were performed by the same operator in a quiet environment after each individual had rested for approximately 15 min. For carotid artery imaging, it was ensured that the neck muscles were relaxed and the patient was lying in a supine position with an angle of approximately 20° towards the neck. Measurements were made from 3 different points 1 cm distal of the right and left anterior carotid artery; only the posterior (distant) wall was evaluated and the intima media thickness measurements were carried out. The average of two measurements was accepted as the mean CIMT.

### 2.5. Statistical analysis

The Shapiro–Wilk test was used to determine whether continuous variables were normally distributed. The Kruskal–Wallis test and Dunn multiple comparison test were used to compare more than two independent groups. The Student t-test was used for the comparison of variables with normal distribution (HDL-C, LDL-C), while the Mann–Whitney U test was used for variables that did not have a normal distribution. Categorical data were compared by chi-square test (sex). The relationships between CIMT, FABP4, Hs-CRP, thyroid function tests (TSH, fT3, fT4, anti-TPO, anti-TG), and metabolic parameters were evaluated by Spearman correlation analysis. The effect of BMI, fT3, and TSH on LDL-C levels was evaluated by multiple regression analysis. Multiple regression analysis was also used to evaluate the effect of BMI and Hs-CRP on FABP4 level and to evaluate the effects of TSH, anti-TPO, TG, and FABP4 on CIMT. SPSS 22.0 for Windows was used for statistical analysis and P < 0.05 was considered statistically significant.

## 3. Results

### 3.1. Demographic, clinical, and biochemical characteristics of participants

Demographic, clinical, and biochemical characteristics of the study groups are shown in Table 1. There was no statistically significant difference in age or sex between the groups (P = 0.198 and P = 0.064, respectively). There was no significant difference between the groups in the levels of creatinine, ALT, HDL-C, F, and HOMA (P = 0.180, P = 0.138, P = 0.900, P = 0.187, P = 0.463, and P = 0.349, respectively). BMI was significantly higher in patients with OH than the healthy controls (P < 0.001). Although BMI was higher in patients with SH compared to healthy controls, this was not statistically significant by a borderline difference (P = 0.06). There was no statistically significant difference in the BMIs of patients with SH and OH.

**Table 1:** Demographic, clinical, and biochemical characteristics of study groups.

	HC	SH	OH	P (HC-SH)	P (HC-OH)	P (SH-OH)
Age (years)	28.0 ± 19.0	32.0 ± 27.0	36.0 ± 12.0	0.312	0.067	0.485
Body weight (kg)	56 ± 7	59 ± 15	64.5 ± 14	0.096	<0.001	0.035
BMI (kg/m2)	21.39 ± 3.52	23.67 ± 5.37	24.4 ± 4.47	0.060	<0.001	0.127
FBG (mg/dL)	94.5 ± 10.0	95.00 ± 10.0	91.0 ± 17.0	0.383	0.593	0.258
Insulin (mU/L)	9.85 ± 4.8	8.0 ± 3.5	8.07 ± 5.0	0.438	0.131	0.228
HOMA	2.12 ± 1.42	1.99 ± 0.9	1.81 ± 1.36	0.535	0.183	0.315
Creatinine (mg/dL)	0.8 ± 0.18	0.82 ± 0.13	0.8 ± 0.2	0.119	0.829	0.1
ALT (mg/dL)	15.0 ± 9.6	14.0 ± 8.8	17.0 ± 9.0	0.927	0.124	0.056
TC (mg/dL)	187.0 ± 51.5	192.0 ± 60.25	198.0 ± 56.5	0.362	0.012	0.177
TG (mg/dL)	79.0 ± 24.0	96.0 ± 75.0	109.5 ± 129	0.004	<0.001	0.101
HDL-C (mg/dL)*	57.0 ± 22.0	54.0 ± 18.0	56.5 ± 22.0	0.598	0.731	1.0
LDL-C (mg/dL)*	109.0 ± 48.0	113.5 ± 40.0	124.5 ± 76.0	0.866	0.045	0.109
fT3 (pg/mL)	3.35 ± 0.6	3.3 ± 0.52	3.0 ± 0.95	0.650	<0.001	<0.001
fT4 (ng/dL)	0.8 ± 0.2	0.8 ± 0.22	0.4 ± 0.3	0.353	<0.001	<0.001
TSH (µIU/mL)	2.12 ± 1.42	1.99 ± 0.9	1.81 ± 1.36	<0.001	<0.001	<0.001
Anti-TPO (U/mL)	2.0 ± 5.0	87.0 ± 381.1	18.0 ± 442.0	<0.001	0.012	0.522
Anti-TG (U/mL)	0.6 ± 2.3	4.1 ± 34.6	0.75 ± 35.8	0.002	0.198	0.161
FABP4 (ng/mL)	5.22 ± 3.29	6.67 ± 6.58	7.03 ± 7.26	0.044	0.014	0.641
Hs-CRP (mg/L)	2.0 ± 3.38	2.7 ± 4.15	3.65 ± 2.85	1.51	0.096	0.491
CIMT (mm)	0.5 ± 0.16	0.5 ± 0.27	0.6 ± 0.2	0.042	<0.001	0.157

Healthy control, HC; subclinical hypothyroidism, SH; overt hypothyroidism, OH; fasting blood glucose, FBG; alanine aminotransferase, ALT; total cholesterol, TC; triglyceride, TG; high-density lipoprotein-cholesterol, HDL-C; low-density lipoprotein-cholesterol, LDL-C; free triiodothyronine, fT3;free thyroxine, fT4; thyroid-stimulating hormone, TSH; antithyroid peroxidase antibodies, Anti-TPO; antithyroglobulin antibodies, Anti-TG; fatty acid binding protein 4, FABP4; high-sensitivity C-reactive protein, Hs-CRP; carotid intima media thickness, CIMT.

In patients with OH, the serum fT3 and fT4 levels were lower than in patients with SH or in HCs, whereas TSH levels were found to be high (P < 0.001 for each comparison). TSH levels were significantly higher in patients with SH than HCs (P < 0.001). There was no significant difference in fT3 and fT4 levels between patients with SH and HCs (P = 0.650 and P = 0.353, respectively). Anti-TPO and anti-TG levels were significantly higher in patients with SH than HCs (P < 0.001 and P = 0.002, respectively). There was no statistically significant difference in anti-TG and anti-TPO levels between patients with SH and OH (P = 0.522 and P = 0.16, respectively).

TG, LDL-C, and TC levels were significantly higher in patients with OH than HCs (P < 0.001, P = 0.045, and P = 0.012, respectively). TG levels were significantly higher in patients with SH when compared to HCs (P = 0.004). There was no significant difference in lipid levels between SH and OH patients (P > 0.05). Although the Hs-CRP level was higher in patients with SH and OH compared to the HC group, it was not statistically significant (P > 0.05).

**Figure 1:**
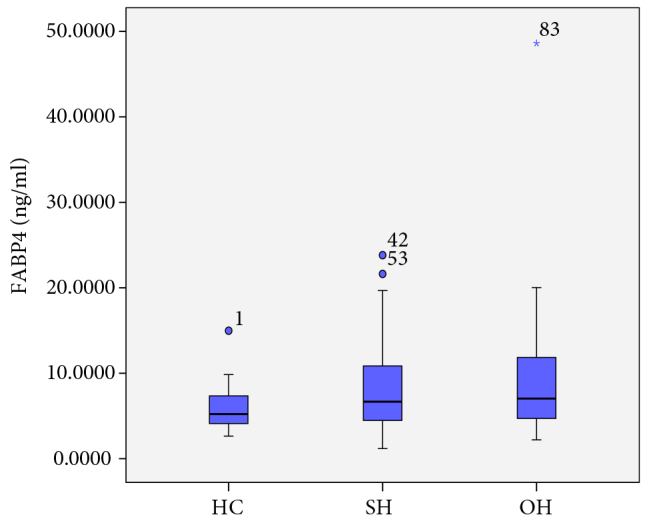
FABP4 levels of groups

**Figure 2:**
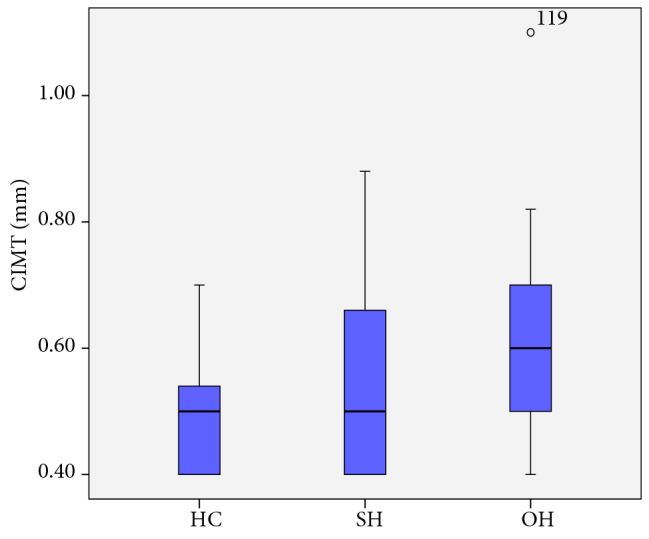
Carotid Intima Media Thickness of Groups

The FABP4 levels of the groups are shown in Figure 1. The FABP4 levels were significantly higher in patients with SH and OH than the HC group (P = 0.044 and P = 0.014, respectively). There was no significant difference in FABP4 levels between SH and OH patients (P = 0.641). In the Spearman correlation analysis, serum FABP4 level was positively correlated with TSH, BMI, and Hs-CRP (r = 0.201, P = 0.039; r = 0.201, P = 0.039; and r = 0.354, P < 0.001, respectively). The correlation graph between FABP4 and Hs-CRP is shown in Figure 2. The independent variables affecting the FABP4 level were evaluated by multiple regression analysis. BMI and Hs-CRP were shown to be correlated with 34.4% of the change in FABP4 level (r = 0.586, r2 = 0.344).

**Table 2:** Multiple regression analysis of variables affecting CIMT.

	β coefficients	P-value
TSH	0.139	0.172
Anti-TPO	0.166	0.093
TG	0.245	0.016
FABP4	0.053	0.601

Thyroid-stimulating hormone, TSH; antithyroid peroxidase antibodies, Anti-TPO; fatty acid binding protein 4, FABP4.r: 0.382, r2: 0.146

The relationships between BMI, metabolic parameters (FBG, insulin, TG, LDL-C, HDL-C, TG, TC), and the parameters related to thyroid function (TSH, fT4, fT3, anti-TPO, anti-TG) were evaluated by Spearman correlation analysis. There was a positive correlation between BMI and LDL-C, TSH, and TC and a negative correlation with fT4 (r = 0.270, P = 0.007; r = 0.311, P = 0.001; r = 0.258, P = 0.01; r = –0.280, P = 0.004). In addition, there was a negative correlation between LDL-C level and fT3 (r = –0.256, P = 0.007). A positive correlation was detected between TG level and TSH and a negative correlation was detected between TG level and fT4 (r = 0.291, P = 0.002; r = –0.282, P = 0.003, respectively). Moreover, a positive correlation was detected between the levels of TC and TSH and a negative correlation was found between fT3 and TC (r = 0.210, P = 0.028; r = –0.282, P = 0.003).

### 3.2. Carotid intima media thicknesses of the participants

**Figure 3:**
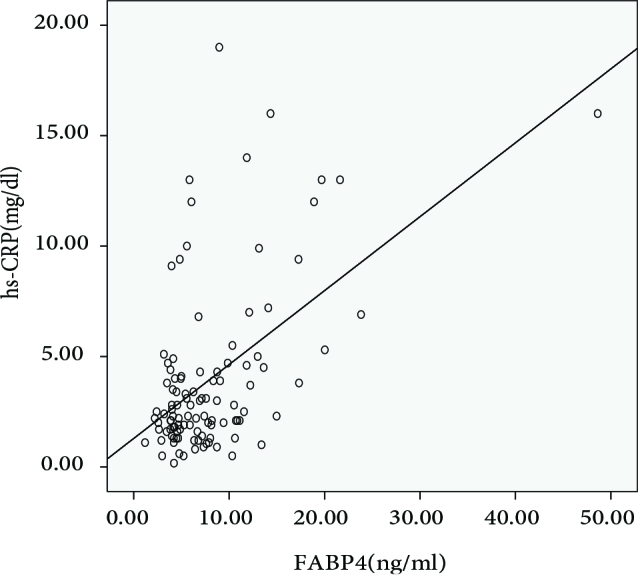
Correlation Between FABP4 and hs-CRP Levels

CIMT was higher in patients with SH and OH compared to the HC group (P = 0.042 and P < 0.001, respectively). The CIMT values of the patients are shown in Figure 3. Spearman correlation analysis indicated a positive correlation between CIMT and TSH, anti-TPO, anti-TG, TG, and TC levels in patients (r = 0.265, P = 0.007; r = 0.251, P = 0.014; r = 0.225, P = 0.028; r = 0.373, P < 0.001; r = 0.252, P = 0.012). No correlation was found between CIMT and FABP4 levels in patients (r = 0.038, P = 0.702). The independent variables affecting CIMT were evaluated by multiple regression analysis. The TG level was found to be an independent factor associated with an increase in CIMT (Table 2).

## 4. Discussion

The most important findings of this study were that CIMT was increased in patients with subclinical and overt hypothyroidism and that the serum level of TG was found to be an independent factor associated with this increase in CIMT. In addition, serum FABP4 levels were found to be increased in patients with SH and OH, but this increase was not correlated with the increase in CIMT.

Increase or decrease in thyroid hormone levels can cause various adverse effects on the cardiovascular system [18]. The etiology for increased risk of CVD in hypothyroid patients includes dyslipidemia, hypercholesterolemia, insulin resistance, endothelial dysfunction, coagulation disorders, inflammation, diastolic HT, and weight gain [18–20].

It is known that elevation of LDL-C has a strong effect in increasing the risk of CVD [21]. In the Helsinki Heart study, even a 7% decrease in LDL-C level was shown to reduce the incidence of CVD by 15% [22]. The most common lipid abnormality in OH is an increase in LDL. Mechanistically, a decrease in LDL receptor level and LDL catabolism has been proposed [21–23]. In addition, elevations of TG and TC are also seen in OH [23]. In the current study, TG, LDL-C, and TC levels were found to be high in patients with OH, which corroborated the published data (P < 0.001, P = 0.045, and P = 0.012, respectively). There was also a positive correlation between TC and the levels of LDL-C and TSH (P = 0.001, P = 0.01, respectively).

Data on lipid alteration in SH are controversial. In the Whickham study (2779 patients, men and women over 18 years of age), no relationship between SH and hyperlipidemia was found; however, the Rotterdam and Nagasaki studies indicated that the TC levels were increased in women with SH [24–26]. Saric et al. reported that TG and LDL levels were higher in patients with SH [27]. 

In a metaanalysis by Abreu et al., the levels of TC and LDL-C were found to be increased in patients with SH and this was correlated with the increase in TSH. These authors also observed a correlation between TG and TSH levels [28]. In the current study, TG level was found to be significantly higher in subjects with SH compared to HCs (P *= *0.004). Differences between the results of various studies may be due to the fact that different cut-off points were used for the definition of SH as well as differences in sex, ethnicity, age, and duration of hypothyroidism in the patient cohorts. Elderly patients are known to be more affected by thyroid dysfunction. In a cross-sectional study with 30,656 participants aged 70–79 years, it was shown that lipid levels were affected in patients whose TSH levels were within the normal range [29]. In a study of 2308 participants by Tognimi et al., a significant impairment in lipid levels was demonstrated in patients with both SH and OH who were older than 65 years [30]. In the current study, the modest effect on lipid levels can be explained by the fact that the patient cohort was relatively young; moreover, these patients did not have any metabolic disorders and their BMIs were within the normal range.

It is well established that a strong relationship exists between circulating inflammatory markers and cardiovascular events. Hs-CRP is commonly used as an inflammatory marker [31]. Serum Hs-CRP levels are known to vary widely in patients with hypothyroidism [12,32,33]. Bell et al. reported that Hs-CRP levels remained unchanged in SH patients [34]. Tuzcu et al. reported that the Hs-CRP level was higher in SH patients and this was also correlated with fasting insulin [35]. Peixoto et al. reported from a study of 12,284 volunteers (11,589 euthyroid, 695 SH) that there was no correlation between TSH and Hs-CRP levels in the SH group and obesity and insulin resistance were the factors responsible for the increase in Hs-CRP in SH patients [11]. In the current study, we did not observe any significant difference in Hs-CRP levels in the participants with OH and SH (P* > *0.05). Additionally, no statistically significant differences were observed for BMI, insulin, and FBG levels between the groups (P *> *0.05). We surmised that the lack of difference in Hs-CRP levels between OH and SH groups could be due to the absence of any metabolic disorder in these patients, which would lead to an increase in inflammation.

Elevation of serum FABP4 is a newly identified risk marker for predicting the development of MetS and CVD [36]. In many studies FABP4 has been shown to play a role in the development of atherosclerosis by activating an inflammatory response through inhibition of endothelial nitric oxide synthetase in smooth muscle cells [37–39]. Both increase in serum FABP4 level and the presence of hypothyroidism have roles in the development of MetS, dyslipidemia, insulin resistance, and CVD [6,8,40–45]. However, the relationship between TSH level and FABP4 level has not been evaluated to date, which we aimed to do in the current study. The FABP4 level was higher in patients with SH and OH compared to HCs (P = 0.044 and P = 0.014, respectively). There was a positive correlation between FABP4 and TSH levels (P = 0.039). However, when multiple regression analysis was performed, we observed that the increase in FABP4 levels could be attributed to increased BMI and inflammation. Although the mean BMI and Hs-CRP levels of the three groups were within the normal ranges, we hypothesized that alterations within the normal range could potentially affect the levels of FABP4. These findings suggest that the reason for the increase in FABP4 levels in hypothyroid patients could be an increase in body weight and inflammation.

Several studies have shown that hypothyroidism increases the risk for the development of CVD [22,46,47]. In a retrospective study by Selmer et al. with 563,700 participants, the risk of CVD and mortality was found to be increased in people with both OH and SH [48]. In addition, Moon et al. carried out a metaanalysis of 35 cohort studies involving 555,530 participants and reported that CVD risk was increased in patients with SH [49]. CIMT measurement has been used in several studies as an early and noninvasive indicator of atherosclerosis [50]. In the current study, CIMT was also used as an indicator of atherosclerosis. It has already been shown that CIMT increases in patients with SH and OH [51]. In the current study, we observed that the increases in CIMT were coupled with increases in TSH levels in patients with both SH and OH. In a multiple regression analysis, it was determined that the independent factor contributing to the increase in CIMT was an increase in TG levels. In addition, no correlation between FABP4 level and CIMT was observed, most likely due to the normal BMIs and absence of any metabolic diseases in the patients. Accordingly, we suggest that there is no effect of FABP4 level on CIMT in hypothyroid patients who do not have any underlying metabolic disorders.

This study has some limitations due to being a cross-sectional study. It would be valuable to demonstrate the changes in thyroid function tests, lipids, and FABP4 levels and the correlations between them after treatment with levothyroxine in patients.

In conclusion, in the current study, an increase in CIMT was observed in patients with SH and OH. In all patients with hypothyroidism, CIMT was more pronounced in those patients with higher TSH levels. In hypothyroid individuals without any metabolic disorders, the independent factor for increasing CIMT was elevation of TG and no effect of FABP4 could be ascertained.

## References

[ref0] (2005). Thyroid-stimulating hormone stimulates interleukin-6 release from 3T3-L1 adipocytes through a cAMP-protein kinase A pathway..

[ref2] (2003). Impaired endothelium-dependent vasodilatation in subclinical hypothyroidism: beneficial effect of levothyroxine therapy. Journal of Clinical Endocrinology &amp; Metabolism.

[ref3] (1997). Flow-mediated, endothelium-dependent vasodilatation is impaired in subjects with hypothyroidism, borderline hypothyroidism, and high-normal serum thyrotropin (TSH) values. Thyroid.

[ref5] (2004). Treating hypothyroidism improves endothelial function. Metabolism.

[ref7] (2007). Endothelial dysfunction as an early sign of atherosclerosis. Herz.

[ref9] (2018). Effects of thyroid-stimulating hormone on adhesion molecules and pro-inflammatory cytokines secretion in human umbilical vein endothelial cells. Research in Pharmaceutical Sciences.

[ref11] (2019). Exercise training–induced changes in metabolic syndrome parameters, carotid wall thickness, and thyroid function in middle-aged women with subclinical hypothyroidism. Pflugers Archiv-European Journal of Physiology.

[ref13] (2000). Subclinical hypothyroidism is an independent risk factor for atherosclerosis and myocardial infarction in elderly women: the Rotterdam Study. Annals of Internal Medicine.

[ref15] (2019). von Haehling S. Subclinical hypothyroidism and the development of heart failure: an overview of risk and effects on cardiac function. Clinical Research in Cardiology.

[ref17] (2018). Thyroid disorders and hemostasis. Seminars in Thrombosis and Haemostasis.

[ref19] (2016). Thyroid function and high-sensitivity C-reactive protein in cross-sectional results from the Brazilian Longitudinal Study of Adult Health (ELSA-Brasil): effect of adiposity and insulin resistance. European Thyroid Journal.

[ref22] (2014). Serum lipids, tHcy, hs-CRP, MDA and PON-1 levels in SCH and overt hypothyroidism: effect of treatment. Acta Biomedica.

[ref24] (2014). Fatty acid-binding protein 4 (FABP4): pathophysiological insights and potent clinical biomarker of metabolic and cardiovascular diseases. Clinical Medicine Insights Cardiology.

[ref26] (2012). and obesity. Turkish Archives of Dermatology and Venereology.

[ref28] (2001). Lack of macrophage fatty-acid–binding protein aP2 protects mice deficient in apolipoprotein E against atherosclerosis. Nature Medicine.

[ref30] (2014). Adipose tissues and thyroid hormones. Frontiers in Physiology.

[ref34] (1998). Correct homeostasis model assessment (HOMA) evaluation uses the computer program. Diabetes Care.

[ref36] (2015). Diagnosis and management of subclinical hypothyroidism in elderly adults: a review of the literature. Journal of American Geriatrics Society.

[ref38] (2003). Hypothyroidism and atherosclerosis. Journal of Clinical Endocrinology &amp; Metabolism.

[ref40] (2012). Prevalence of cardiovascular diseases in patients with hypothyroidism. Revista Medico-Chirurgicala a Societatii de Medici si Naturalisti din Iasi.

[ref42] (1994). Triglyceride concentration and coronary heart disease. British Medical Journal.

[ref44] (1998). Lipid alterations and decline in the incidence of coronary heart disease in the Helsinki Heart Study. Journal of the American Medical Association.

[ref46] (2000). Cardiovascular and atherogenic aspects of subclinical hypothyroidism. Thyroid.

[ref49] (1977). The spectrum of thyroid disease in a community: the Whickham survey. Clinical Endocrinology.

[ref51] (2010). The incidence of ischemic heart disease and mortality in people with subclinical hypothyroidism: reanalysis of the Whickham Survey cohort. Journal of Clinical Endocrinology &amp; Metabolism.

[ref53] (2005). Decrease of arterial stiffness at common carotid artery in hypothyroid patients by normalization of thyroid function. Biomedicine &amp; Pharmacotherapy.

[ref55] (2017). Dyslipidemia in subclinical hypothyroidism requires assessment of small dense low density lipoprotein cholesterol (sdLDL-C). Romanian Journal of Internal Medicine.

[ref57] (2017). Subclinical hypothyroidism: to treat or not to treat, that is the question! A systematic review with meta-analysis on lipid profile. Endocrine Connections.

[ref59] (2002). Association between thyroid dysfunction and total cholesterol level in an older biracial population: the health, aging and body composition study. Archives of Internal Medicine.

[ref61] (2012). Age and gender substantially influence the relationship between thyroid status and the lipoprotein profile: results from a large cross-sectional study. Thyroid.

[ref63] (2018). C-reactive protein in atherothrombosis and angiogenesis. Frontiers in Immunology.

[ref65] (2004). Serum high sensitivity C-reactive protein is associated with carotid intima-media thickness in type 2 diabetes. Diabetes Research and Clinical Practice.

[ref67] (2014). Dyslipidemia associated with subclinical hypothyroidism in Eastern Nepal. American Journal of the Medical Sciences.

[ref70] (2007). related quality of life and cardiovascular disease risk profile in women with subclinical thyroid disease–a community‐based study. Clinical Endocrinology.

[ref72] (2005). be associated with elevated high-sensitive c-reactive protein (low grade inflammation) and fasting hyperinsulinemia. Endocrine Journal.

[ref75] (2018). Stearic acid induces CD11c expression in proinflammatory macrophages via epidermal fatty acid binding protein. Journal of Immunology.

[ref77] (2012). Fatty acid-binding protein 4 impairs the insulin-dependent nitric oxide pathway in vascular endothelial cells. Cardiovascular Diabetology.

[ref79] (2011). Chronic administration of BMS309403 improves endothelial function in apolipoprotein E‐deficient mice and in cultured human endothelial cells. British Journal of Pharmacology.

[ref82] (2009). Endothelial dysfunction in aged humans is related with oxidative stress and vascular inflammation. Aging Cell.

[ref84] (2018). Endothelial dysfunction and the risk of atherosclerosis in overt and subclinical hypothyroidism. Endocrine Connections.

[ref86] (2018). Novel transcriptional mechanisms for regulating metabolism by thyroid hormone. International Journal of Molecular Sciences.

[ref88] (2012). Elevation of fatty acid-binding protein 4 is predisposed by family history of hypertension and contributes to blood pressure elevation. American Journal of Hypertension.

[ref90] (2006). Adipocyte fatty acid–binding protein is a plasma biomarker closely associated with obesity and metabolic syndrome. Clinical Chemistry.

[ref92] (2013). Elevated circulating adipocyte-fatty acid binding protein levels predict incident cardiovascular events in a community-based cohort: a 12-year prospective study. Journal of the American Heart Association.

[ref94] (2007). Treatment of diabetes and atherosclerosis by inhibiting fatty-acid-binding protein aP2. Nature.

[ref97] (2000). Cardiovascular and atherogenic aspects of subclinical hypothyroidism. Thyroid.

[ref99] (2005). Subclinical thyroid dysfunction as a risk factor for cardiovascular disease. Archives of Internal Medicine.

[ref101] (2014). Subclinical and overt thyroid dysfunction and risk of all-cause mortality and cardiovascular events: a large population study. Journal of Clinical Endocrinology &amp; Metabolism.

[ref103] (2018). Subclinical hypothyroidism and the risk of cardiovascular disease and all-cause mortality: a meta-analysis of prospective cohort studies. Thyroid.

[ref105] (1999). Carotid plaque, intima media thickness, cardiovascular risk factors, and prevalent cardiovascular disease in men and women: the British Regional Heart Study. Stroke.

[ref108] (2010). Carotis intima media thickness in female patiens with subclinical hypothyroidism. Turkish Journal of Endocrinology and Metabolism.

